# Effect of Radiant Catalytic Ionization and Ozonation on *Salmonella* spp. on Eggshells

**DOI:** 10.3390/foods11162452

**Published:** 2022-08-14

**Authors:** Katarzyna Grudlewska-Buda, Natalia Wiktorczyk-Kapischke, Ewa Wałecka-Zacharska, Joanna Kwiecińska-Piróg, Grzegorz Gryń, Karolina Jadwiga Skowron, Jakub Korkus, Eugenia Gospodarek-Komkowka, Jarosław Bystroń, Anna Budzyńska, Stefan Kruszewski, Zbigniew Paluszak, Małgorzata Andrzejewska, Monika Wilk, Krzysztof Skowron

**Affiliations:** 1 Department of Microbiology, Nicolaus Copernicus University in Toruń, Ludwik Rydygier Collegium Medicum, 85-094 Bydgoszcz, Poland; 2 Department of Food Hygiene and Consumer Health, Wrocław University of Environmental and Life Sciences, 50-375 Wrocław, Poland; 3 Plant Breeding and Acclimatization Institute–National Research Institute, 85-090 Bydgoszcz, Poland; 4 Institute of Telecommunications and Computer Science, Jan and Jędrzej Śniadecki University of Technology in Bydgoszcz, 85-094 Bydgoszcz, Poland; 5 Biophysics Department, Nicolaus Copernicus University in Toruń, Ludwik Rydygier Collegium Medicum, 85-067 Bydgoszcz, Poland; 6 Department of Microbiology and Food Technology, Jan and Jędrzej Śniadecki University of Technology in Bydgoszcz, 85-094 Bydgoszcz, Poland; 7 Department of Hygiene, Epidemiology, Ergonomy and Postgraduate Education, Ludwik Rydygier Collegium Medicum in Bydgoszcz, Nicolaus Copernicus University in Toruń, 95-094 Bydgoszcz, Poland

**Keywords:** eggs, *Salmonella* spp., radiant catalytic ionization, ozonation, disinfection

## Abstract

Three *Salmonella enterica* strains were used in the study (serovars: *S. enteritidis*, *S. typhimurim* and *S. virchow*). This study evaluated the efficacy of radiant catalytic ionization (RCI) and ozonation against *Salmonella* spp. on eggshell (expressed as log CFU/egg). The egg surface was contaminated three different bacterial suspension (10^3^ CFU/mL, 10^5^ CFU/mL and 10^8^ CFU/mL) with or without poultry manure. Experiments were conducted at 4 °C and 20 °C in three different time period: 30 min, 60 min and 120 min. Treatment with RCI reduced *Salmonella* numbers from 0.26 log CFU/egg in bacterial suspension 10^8^ CFU/mL, 4 °C and 20 °C, with manure for 30 min to level decrease in bacteria number below the detection limit (BDL) in bacterial suspension 10^5^ CFU/mL, 20 °C, with or without manure for 120 min. The populations of *Salmonella* spp. on eggs treated by ozonizer ranged from 0.20 log CFU/egg in bacteria suspension 10^8^ CFU/mL, 20 °C, with manure for 30 min to 2.73 log CFU/egg in bacterial suspension 10^5^ CFU/mL, 20 °C, with manure for 120 min. In all treatment conditions contamination with poultry manure decrease effectiveness the RCI and ozonation. In summary, RCI technology shows similar effectiveness to the ozonation, but it is safer for poultry plant workers and consumers.

## 1. Introduction

For many years *Salmonella* spp. has been one of the most important foodborne pathogens. The European Food Safety Agency (EFSA), in 2018, reported 91,857 confirmed cases of salmonellosis in the European Union (EU). The most prevalent serovars were: *S. enteritidis*, *S. typhimurium* and *S*. Infantis [1]. The significant source of human infections are eggs and egg products, which in 2018 accounted for 45.6% of salmonellosis foodborne outbreaks [1]. A multi-country outbreak of *S. enteritidis*, linked to eggs, has been ongoing in the EU for several years. From 1 February 2017 to 14 January 2020, 15 EU countries reported 656 confirmed cases and 202 probable cases [2].

There are two possible routes of bacterial contamination of eggs shell: either vertically or horizontally. Horizontal transmission occurs during the laying of eggs and depending on the eggshell architecture and bacterial serotype. Vertical transmission can originate from the hen reproductive tract. *S. enteritidis* is usually transmitted vertically, while *S*. Infantis contaminates the egg via a horizontal route [3]. Minor flaws of the eggshell favor bacterial colonization and transmission [4]. Bacterial contamination of eggshells depends on various environmental factors such as the presence of food, water, feces, dust, litter, the type of birds’ housing system, the laying rate and/or cuticle state [5]. The thickness of particular layers, pore distribution, ultrastructure and transparency affect the eggshell penetration [6]. Furthermore, workers, domestic animals, rodents, contaminated feed, litter and water [7] or food production environment (transfer belt, packaging materials) can be the source of eggs contamination [8].

Cleaning and disinfection are the most common methods used to remove microbiological contamination from the egg surface [9]. Cleaning with chemical agents, e.g., alkaline solutions or sodium hydroxide removes the cuticle layer resulting in a visually clean egg [10]. Cleaning significantly reduced the number of Enterobacterales bacteria [11]. Regularly applied disinfectants include agents based on chlorine, iodine, hydrogen peroxide, ozone and quaternary ammonium compounds [12].

Methods of egg disinfection must limit the growth of microorganisms outside the shell and limit the penetration of microorganisms into the inside of the egg [13]. The ozonation may use for the disinfection of hatcheries, eggs and poultry carcasses. Ozone has a good bactericidal effect and causes a quick inactivation of microorganisms. The effectiveness of ozonation increases with the extension of the exposure time of the eggs. It is related to the increase in ozone concentration over time [14]. The hydrophobic protein layer (cuticle) hinders bacterial penetration. This thin outermost layer desiccates immediately after eggs laying and protects eggs against bacterial invasion and water loss [15].

Researchers are still searching for innovative technologies allowing the eradication of pathogens from the egg surface. Such methods should eliminate microbiological contaminants ensuring egg freshness and consumer safety. New technologies include physical and chemical processes such as high hydrostatic pressure, ionizing radiation, ultrasounds, pulsed electric field, UV radiation and plasma, which inactivate microorganisms at ambient or sublethal temperatures [16]. Additionally, some preparations are applied directly to the eggshell, e.g., colloidal silver, substances of natural origin (propolis) or plant extracts such as thyme and cinnamon, allicin, oregano oil or red grapefruit juice [17]. An innovative solution, successfully applied in the air purification system, is radiant catalytic ionization (RCI) [18]. This technology uses the photocatalysis phenomenon in the presence of UV radiation and photocatalysts, such as TiO_2_, which form a hydrophilic coating of the matrix surface in the RCI module [19]. The RCI cell consists of matrices forming a honeycomb structure. The coating of the dies also includes clusters of other elements such as rhodium, silver and copper. On the opposite site a broad-spectrum UV light source of 100 and 367 nm is located [19,20]. Catalytic oxidation, stimulated by UV radiation, at the boundary of heterogeneous phases (gas-solid), leads to reactive oxygen forms generation (ROS): hydroxyl radicals (OH^•^), hydrogen peroxide (H_2_O_2_) and superoxide anion (O^2−^). The total number of generated ions is about 5.0 × 10^4^ ions × cm^−3^ of air [21]. ROS interact with DNA, lipids and proteins, contributing to the destruction of genetic material, lipid peroxidation and amino acid degradation [22,23,24].

To date, RCI has been used primarily in the air purification industry. The method successfully eliminated biofilm and planktonic cells from various surfaces, indicating its applicability for disinfecting food processing surfaces and healthcare equipment [21]. Studies by Ortega et al. [23] and Skowron et al. [25] have demonstrated the utility of using RCI against various pathogens, including *Salmonella* spp. from different surfaces. The discussed technology has also proven effective when removing biofilms from abiotic surfaces contaminated with food pulp [21] and from vegetable and fruit surfaces [22], suggesting its possible application in food disinfection.

This study aimed to assess and compare the efficacies of radiant catalytic ionization (RCI) and ozonation against *Salmonella* spp. on the eggshell, with different initial inocula, exposure time and processing temperature.

## 2. Materials and Methods

### 2.1. Bacterial Strains

The study was conducted on 3 *Salmonella enterica* strains isolated in 2017 from poultry meat, representing different serovars: *S. enteritidis*, *S. typhimurim* and *S. virchow* (most commonly identified salmonellosis serovars in Poland and Europe). Study strains represented different phage types (DTs) (*S. enteritidis*–DT8 (commonly associated with consumption of eggs), *S. typhimurim*–DT2), for *S.* Virchow phage type could not be determined.

### 2.2. Eggshells Contamination with Salmonella spp.

Fresh eggs of class M (medium eggs, with weight ranging from 53 to 63 g) were wiped with a fine damp cloth to remove visible soil. Next, eggs were sterilized with a high-energy electron beam (20 kGy) in the Institute of Nuclear Chemistry and Technology in Warsaw, Poland. The efficacy of disinfection was confirmed by taking swabs from the egg surface and plating them onto Columbia Agar with 5% blood sheep (bioMérieux, Marcy-l’Etoile, France) (37 °C, 24 h).

This study used bacterial suspensions of three different densities, 10^8^ CFU/mL (ranged from 8.43 log CFU/mL to 8.60 log CFU/mL), 10^5^ CFU/mL (ranged from 5.68 log CFU/mL to 5.76 log CFU/mL) and 10^3^ CFU/mL (ranged from 2.70 log CFU/mL to 2.83 log CFU/mL), representing heavy, medium and light contamination, respectively. This step was used to establish the initial number of bacteria in prepared suspensions with which the eggs were contaminated, therefore the number is referred to as CFU/mL.

The 0.5 McFarland’s bacterial suspension (8.43 log to 8.60 log CFU/mL) was prepared as 10^8^ CFU/mL (initial bacterial contamination level). Then, this initial suspension was diluted to obtain the inoculum of 10^3^ or 10^5^ CFU/mL. Another variant of the experiment was the contamination of the egg with organic pollution. The microbial suspensions were mixed in a volumetric ratio (v/v) 1:1 with fresh poultry manure (from a hen farm in Poland, collected in March 2021). Poultry manure was stored at 4 °C until testing.

Each side of the egg surface was contaminated with hundred drops (5 µL each) of bacterial suspension and dried (20 min in a laminar chamber (1 m^3^) per one side of the egg). The drops did not flow freely down the egg. Experiments were conducted at 4 °C (fridge, standard size) and 20 °C (hermetic chamber, 1 m^3^). We used 324 eggs for each strain, including all variants and repetitions.

**Figure 1:**
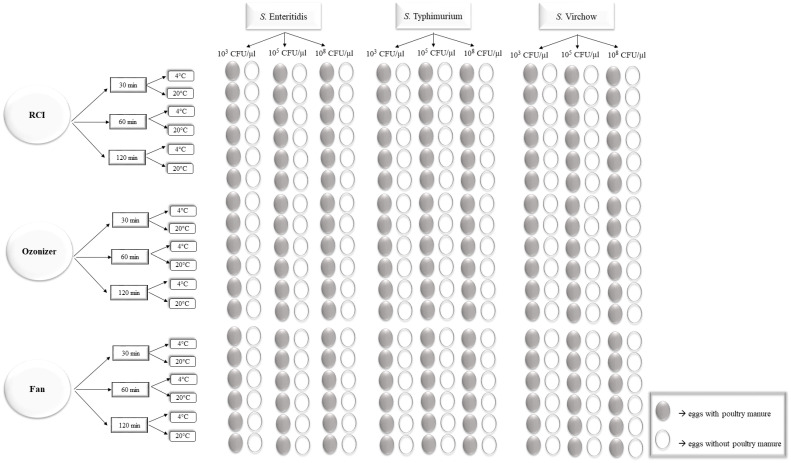
Scheme of experiment variants.

Figure 1 shows the design of the experiment.

### 2.3. Exposure of Salmonella spp. Contaminated Eggshells to Radiant Catalytic Ionization and Ozonation

**Figure 2:**
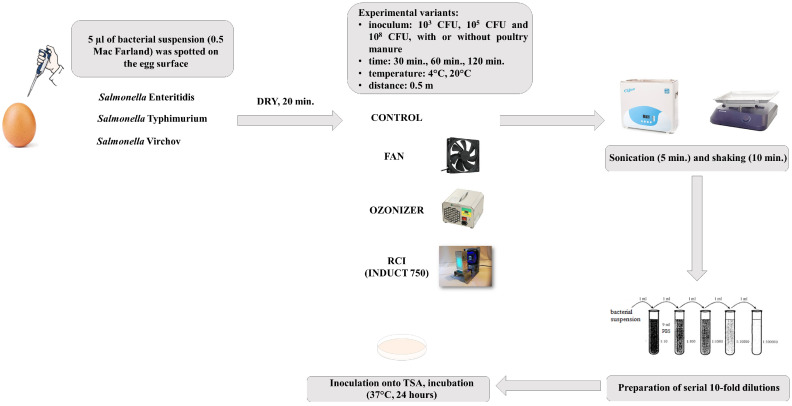
A scheme of experiment procedure.

The eggs contaminated with bacterial suspension were placed on a wire stand (which allowed the device to operate evenly on the egg) at a distance of 0.5 m from Induct 750 ActiveTek apparatus (Kielce, Poland) (not generating ozone), Dawid 2 ozonizer (ECS Piotr Paruszewski, Ostrzeszów, Poland) (generating 10 g/h O_3_) or the fan (control treatment) and exposed for 30 min, 60 min or 120 min to RCI, ozone or the flowing air, respectively. To determine the number of bacteria reisolated from eggshells, eggs were placed in sterile plastic containers with closures filled with 100 mL of sterile saline (Avantor, Gliwice, Poland). Next, they were sonicated (Ultrasonic DU-4 sonicator, Nickel-Electro, Oldmixon, Great Britain) for 5 min and shaken (400 rpm) for 10 min (benchtop shaker). Three non-treated with RCI or ozone eggs allowed determining the initial number of bacteria reisolated after contamination of eggshells with tested suspensions. After sonication, serial 10-fold dilutions in sterile saline for each bacterial suspension obtained after sonication and shaking were prepared. The enumeration was performed on tryptic soy agar (TSA) (bioMerieux). After 24-h incubation at 37 °C the number of colonies per egg was calculated (log CFU/egg) (Figure 2). The procedure included three repetitions for each tested strain and each variant.

Decrease in the number of bacteria (DB) (log CFU/egg) after treatment was calculated using the formula: DB = A − B

Where:

A—initial number of bacteria [log CFU/egg];

B—number of bacteria after treatment [log CFU/egg].

### 2.4. Statistical Analysis

Each experiment was repeated three times. A multivariate ANOVA and the Tukey post-hoc test with Statistica (TIBCO Software Inc., Palo Alto, CA, USA) were performed to determine whether statistical differences existed between different experimental groups. Significance was set at a level of *p* ≤ 0.05.

## 3. Results

In our experiment, differences between strains were not statistically significantly different (*p* > 0.05). Therefore, we decided to average the results for all serovars. Detailed results regarding the changes of the bacterial number of the tested *Salmonella* spp. serovars in the bacterial suspension of 10^3^ CFU/mL, 10^5^ CFU/mL and 10^8^ CFU/mL were included in the Supplementary Information (Tables S1–S3).

### 3.1. Effectiveness of Radiant Catalytic Ionization and Ozonation Treatment against Salmonella spp. for Bacterial Suspension of 10^3^ CFU/mL

The bacterial number reisolated from the eggshell in the control group (not exposed to any technology) ranged from 1.59 log CFU/egg to 1.67 log CFU/egg, and was not significantly different (*p* > 0.05) (Table 1). After 30 min of exposure, the bacterial number ranged from 0.03 log CFU/egg (RCI, 20 °C, without poultry manure) to 0.69 log CFU/egg (ozonizer, 20 °C, with poultry manure). Statistically significant differences were observed in all bacterial contamination level (4 °C with poultry manure, 20 °C without poultry manure, 20 °C with poultry manure) (Table 1). The 60-min and 120-min application of ozonizer and RCI-emitter decreased the bacteria number to detection limit, which is <100 CFU/egg.

Treatment with RCI reduced bacteria number ranged from 1.39 log CFU/egg (30 min, 4 °C, with poultry manure) to level decrease in bacteria number below the detection limit (BDL) (60 min and 120 min, 4 °C and 20 °C, without and with poultry manure). For ozonizer reduction in the number of bacteria ranged from 0.98 log CFU/egg (30 min, 20 °C, with poultry manure) to BDL (60 min and 120 min, 4 °C and with poultry manure, 20 °C and without poultry manure, 20 °C and with poultry manure) (Table 2).

The bacterial number after 60 min and 120 min of fan action was 0.00 log CFU/egg. However, a shorter time of exposure (30 min) reduced the number of bacteria from 0.57 log CFU/egg (20 °C and without poultry manure) to 0.98 log CFU/egg (20 °C and with poultry manure). The reduction was significantly different (*p* < 0.05) lower compared to RCI-exposure for the same time in all variants.

### 3.2. Effectiveness of Radiant Catalytic Ionization and Ozonation Treatment against Salmonella spp. for Bacterial Suspension of 10^5^ CFU/mL

The differences in the control group were not statistically significant (*p* > 0.05) (Table 3). The bacterial number reisolated from the eggshell ranged from 2.65 log CFU/egg to 2.75 log CFU/egg. The application of ozonizer and RCI-emitter reduced bacterial number. After 30 min-exposure, the bacterial number ranged from 1.29 log CFU/egg (RCI, 20 °C and without poultry manure) to 2.48 log CFU/egg (ozonizer, 20 °C and with poultry manure). There were no statistically significantly differences between both methods.

After 60 min-exposure, the bacterial number ranged from 0.07 log CFU/egg (RCI, 20 °C and without poultry manure) to 2.36 log CFU/egg (ozonizer, 4 °C and with poultry manure). Statistically significant differences (*p* ≤ 0.05) were found between RCI at 20 °C without poultry manure and fan under all conditions, and ozonizer at 4 °C and 20 °C with poultry manure and RCI at 4 °C and 20 °C with poultry manure.

After 120 min. of exposure, the lowest number of *Salmonella* spp. was noted for RCI at 20 °C with or without poultry manure (0.00 log CFU/egg) and for ozonizer at 20 °C with and without poultry manure. These values were statistically different (*p* ≤ 0.05) from all methods at 4 °C with poultry manure or fan at 20 °C with poultry manure (Table 3).

For treatment with RCI bacterial number ranged from 0.30 log CFU/egg (30 min, 4 °C, with poultry manure) to BDL (120 min, 20 °C, without and with poultry manure). For ozonizer bacterial reduction ranged from 0.26 log CFU/egg (30 min, 20 °C, with poultry manure) to 2.73 log CFU/egg (120 min, 20 °C, with poultry manure) (Table 2).

The bacterial number after fan action ranged from 0.96 log CFU/egg (120 min, 4 °C, without poultry manure) to 2.62 log CFU/egg (30 min, 20 °C, with poultry manure). Moreover, reduction number of bacteria for the fan were the lowest and ranged from 0.13 log CFU/egg (30 min, 4 °C and 20 °C, with poultry manure) to 1.70 log CFU/egg (120 min, 4 °C, without poultry manure).

### 3.3. Effectiveness of Radiant Catalytic Ionization and Ozonation Treatment against Salmonella spp. for Bacterial Suspension of 10^8^ CFU/mL

The number of reisolated *Salmonella* spp. in the control group ranged from 5.81 log CFU/egg to 5.89 log CFU/egg, but differences were not statistically significant (*p* > 0.05). (Table 4). In all time variants, the number of reisolated bacteria was higher than at the concentration of 10^5^ CFU/mL.

After 30 min of exposure, the bacterial number ranged from 4.83 log CFU/egg (RCI, 4 °C, without poultry manure) to 5.69 log CFU/egg (ozonizer, 20 °C, with poultry manure). After RCI-emitter and ozonizer treatment at both tested temperatures, the number of bacteria was significantly different higher with the poultry manure (*p* ≤ 0.05) (Table 4).

After 60 min of exposure, the bacterial number ranged from 3.69 log CFU/egg (RCI, 20 °C, without poultry manure) to 5.57 log CFU/egg (ozonizer, 20 °C, with poultry manure).

After 120 min of exposure, the lowest number of bacteria was found for RCI at 20 °C without poultry manure (2.27 log CFU/egg). The value was significantly different (*p* ≤ 0.05) from the value for the ozonizer under the same experimental conditions. The ozonizer treatment at 20 °C with poultry manure caused the lowest reduction (5.40 log CFU/egg). In all time variants, at both temperatures RCI and ozonizer reduced statistically significantly higher the bacterial number in the absence of poultry manure (Table 4).

RCI treatment caused a decline of bacteria ranged from 0.26 log CFU/egg (30 min, 4 °C and 20 °C, with poultry manure) to 3.54 log CFU/egg (120 min, 20 °C, without poultry manure). For ozonizer bacterial reduction ranged from 0.20 log CFU/egg (30 min, 20 °C, with poultry manure) to 1.91 log CFU/egg (120 min, 4 °C, without poultry manure) (Table 2). RCI treatment statistically significantly higher (*p* ≤ 0.05) reduce of *Salmonella* spp. number in bacterial contamination level 10^8^ CFU/mL in 60 min and 120 min-exposure, 20 °C, without poultry manure than other tested methods.

## 4. Discussion

Commonly used methods for egg disinfection include chemical agents, but adverse effects of high temperature on, e.g., fatty acids, vitamins or cholesterol, are widely known. However, increased consumers’ awareness forced the researchers to search for new methods, effective against microbes and safe for the environment and public health. New technologies such as RCI can reduce the use of disinfectants, but are not intended to completely replace them. The combined use of different physical and chemical methods results in increased microbiological safety of eggs. RCI technology, for maximum effectiveness, requires adequate working time, the right time of contact of active air with egg shells and the appropriate distance [26]. Such devices could be placed directly in laying hen houses or in egg stores or in hatching apparatuses. Then the working time of the technology could be sufficiently long. This technology is safe for humans and animals and is designed for continuous operation. Research conducted on the influence of RCI technology on hatchability of eggs, weight of chicks and occurrence of developmental defects did not show significant differences compared to the control variant without the use of this technology (unpublished data).

In the current study, we determined the effect of RCI and ozonation on the eggshells contaminated with *Salmonella* spp. Both methods significantly reduced the bacterial number of all tested serotypes, but their effectiveness varied depending on the variant used.

The initial bacterial contamination seems to play a crucial role in the efficacy of tested technology. However, our study shows that both methods are efficient even at high bacterial density. In low bacterial contamination level (10^3^ CFU log/egg) and shorter time exposure (30 min) RCI-technology was more effective, suggesting its application as the disinfection method in egg processing plants. Soljour et al. [27] indicated that sodium carbonate, sodium hypochlorite and potassium hydroxide applied at the recommended concentrations eliminate *S. enteritidis* from eggshells contaminated with 10^4^ or 10^6^ CFU/mL of the bacterial suspension.

Moreover, temperature, organic solution and exposure time of RCI or ozone largely affect the decontamination power. Our previous research showed the lowest reduction of bacteria number for surfaces contaminated with meat and fish pulp before the action of RCI (24 h, 0.5 m, 20 °C). The reduction rate was equal to 0.89 log CFU × cm^–2^ for *Staphylococcus aureus*, 1.17 log CFU × cm^–2^ for *Listeria monocytogenes*, 1.43 log CFU × cm^–2^ for *S. enteritidis* and 1.61 log CFU × cm^–2^ for *Escherichia coli* O157:H7 [26]. The influence of poultry manure on the survival of bacteria was noticeable in these studies. Each time their addition decreased the effectiveness of the tested methods and increased the survival rate of *Salmonella* spp. Furthermore, Bing et al. [28] showed that feces protect bacteria on eggshells against the bactericidal effects of other methods, e.g., UV-C radiation.

In most cases, the effectiveness of RCI was higher than that of ozonation, but usually, these differences were not statistically significant. Mannozzi et al. [22] indicated the reduction of bacteria (*E. coli*, *S. typhimurium* and *Listeria innocua*) from the surface of apple peel and spinach leaves after 90 min of exposure to RCI. For cantaloupe researchers obtained reductions of 94% and 88% for *E. coli* and *S. typhimurium*, respectively. The effectiveness of the RCI was influenced by the operating time of the device and the type of surface [22].

In our study, the treatment with ozone (10 g/h) reduced bacterial number on the eggshell. This number decreased with the exposure time extension, in both variants of the bacterial suspension density and at both temperatures. On the contrary, Braun et al. [14] have reported complete inactivation of *S. enteritidis* (contamination level of 10^2^–10^4^ CFU/g) on the eggshell after 120 min treatment with 1% ozone. In turn, Rodriguez-Romo and Yousef [29] applying ozonation for 10 min (15 lb/in2 [103.421 kPa], 4 to 8°C) reduced up to 5.9 log CFU/g of *S. enteritidis*. The discrepancy between our research and the studies discussed above can result from the different strains, experimental conditions and ozone concentration used. A fundamental aspect of the use of ozone for egg disinfection is its safety. Wlazlo et al. [17] have demonstrated that ozone treatment reduced hatching of eggs and significantly increased egg mortality. This finding may indicate the negative impact of this gas on developing embryos. Some studies indicated that exposure of hen eggs to ozone deteriorated the nutritive characteristics of the eggs (low amount of yolk tocols, carotenoids, cholesterol and lipid oxidative status) and lowered eggshell breaking strength [30,31].

The conducted study has a several limitations that should be supplemented in further studies. In next experiments, it is worth considering other bacteria that may also be present on the shells of hens’ eggs (e.g., *E. coli*, *Campylobacter* spp., *Staphylococcus* spp., *Streptococcus* spp., *Yersinia* spp. and *L. monocytogenes*). It is also worth considering shorter exposure times, e.g., a few minutes. If satisfactory efficiency were demonstrated, the technology could be applied at stages other than egg storage. Valuable information would also be provided by the study of hatching eggs, including the impact of RCI technology on their hatchability as well as the health and survival of chicks.

## 5. Conclusions

In conclusion, a constant urge to meet the consumer expectations of fresh, microbiologically safe food products results in the search for new disinfection methods. The findings of the present study indicate that the most effectiveness treatment was RCI and ozonation at contamination level 10^3^ CFU/mL especially with treatment duration of 60 and 120 min. However, at the higher initial contamination levels of 10^5^ and 10^8^, the effectiveness of RCI and ozonation was also high with longer treatment. The addition of poultry manure reduced the effectiveness of both methods each time. Because of RCI effectively eliminates *Salmonella* spp. on the eggshells this technology may be a good candidate for the enhancement of biosecurity at farms and egg processing plants.

## Figures and Tables

**Table 1:** The final number of *Salmonella* spp. on eggshells contaminated with bacterial suspension of 10^3^ CFU/mL with or without the addition of poultry manure.

Variant		Temperature 4 °C		Temperature 20 °C	
		PM (−)	PM (+)	PM (−)	PM (+)
		Average [log CFU/egg] (±STD) *	Average [log CFU/egg] (±STD)	Average [log CFU/egg] (±STD)	Average [log CFU/egg] (±STD)
Control		1.59 ^h^ (±0.09)	1.67 ^h (^±0.09)	1.59 ^h^ (±0.09)	1.67 ^h^ (±0.09)
30 min ^†^	Fan	0.58 ^e,f^ (±0.02)	0.96 ^g^ (±0.02)	0.57 ^d,e,f^ (±0.00)	0.98 ^g^ (±0.02)
	Ozonizer	0.34 ^b,c,d,e^ (±0.04)	0.61 ^e,f^ (±0.02)	0.41 ^c,d,e,f (^±0.02)	0.69 ^f,g^ (±0.01)
	RCI	0.09 ^a,b^ (±0.07)	0.28 ^a,b,c,d^ (±0.05)	0.03 ^a^ (±0.09)	0.20 ^a,b,c^ (±0.06)

*—standard deviation, ^†^—time of action; PM (+)—with poultry manure; PM (−)—without poultry manure CFU–colony forming units; ^a,b,c,…^—values marked with different letters differ statistically significant, applies to the entire table.

**Table 2:** Decrease in the number of bacteria [log CFU/egg] on eggshells after treatment.

Variant			Temperature 4 °C		Temperature 20 °C	
			PM (−)	PM (+)	PM (−)	PM (+)
Bacterial suspension 10^3^ CFU	30 min ^†^	Fan	1.01 ^h,i,j,k,l,ł,m,n^	0.71 ^d,e,f,g,h,i^	1.01 ^h,i,j,k,l,ł,m,n^	0.69 ^c,d,e,f,g,h,i^
		Ozonizer	1.24 ^k,l,ł,m,n,o,p^	1.05 ^h,i,j,k,l,ł,m,n^	1.17 ^i,j,k,l,ł,m,n,o^	0.98 ^h,i,j,k,l,ł,m^
		RCI	1.50 ^n,o,p,r,s^	1.39 ^ł,m,n,o,p,r^	1.56 ^o,p,r,s,t^	1.47 ^m,n,o,p,r,s^
	60 min	Fan	BDL *	BDL	BDL	BDL
		Ozonizer	BDL	BDL	BDL	BDL
		RCI	BDL	BDL	BDL	BDL
	120 min	Fan	BDL	BDL	BDL	BDL
		Ozonizer	BDL	BDL	BDL	BDL
		RCI	BDL	BDL	BDL	BDL
Bacterial suspension 10^5^ CFU	30 min †	Fan	0.38 ^a,b,c,d,e,f,g^	0.13 ^a,b^	0.38 ^a,b,c,d,e,f,g^	0.13 ^a,b^
		Ozonizer	1.12 ^i,j,k,l,ł,m,n,o^	0.28 ^a,b,c,d,e,f^	0.97 ^a^	0.26 ^a,b,c,d,e^
		RCI	1.28 ^l,ł,m,n,op^	0.30 ^a,b,c,d,e,f^	1.37 ^ł,m,n,o,p,r^	0.32 ^a,b,c,d,e,f^
	60 min	Fan	0.76 ^f,g,h,i,j,k^	0.21 ^a,b,c^	0.74 ^e,f,g,h,i,,j^	0.21 ^a,b,c^
		Ozonizer	2.00 ^t,u^	0.39 ^a,b,c,d,e,f,g^	1.83 ^r,s,t,u^	1.17 ^i,j,k,l,ł,m,n,o^
		RCI	1.95 ^s,t,u^	0.40 ^a,b,c,d,e,f,g^	2.59 ^w,x^	1.22 ^j,k,l,ł,m,n,o,p^
	120 min	Fan	1.70 ^p,r,s^.^t,u^	0.37 ^a,b,c,d,e,f,g^	1.36 ^ł,m,n,o,p,r^	0.36 ^a,b,c,d,e,f,g^
		Ozonizer	2.14 ^u,w^	0.59 ^b,d,e,f,g,h^	2.64 ^x,y^	2.73 ^y^
		RCI	2.15 ^u,w,x^	0.59 ^b,d,e,f,g,h^	BDL	BDL
Bacterial suspension 10^8^ CFU	30 min †	Fan	0.32 ^a,b,c,d,e,f^	0.09 ^a^	0.32 ^a,b,c,d,e,f^	0.09 ^a^
		Ozonizer	0.94 ^h,i,j,k,l,ł^	0.22 ^a,b,c,d^	0.85 ^g,h,i,j,k,l^	0.20 ^a,b,c^
		RCI	0.98 ^h,i,j,k,l,ł,m^	0.26 ^a,b,c,d,e^	1.05 ^h,i,j,k,l,ł,m,n^	0.26 ^a,b,c,d,e^
	60 min	Fan	0.60 ^b,d,e,f,g,h^	0.19 ^a,b,c^	0.59 ^b,d,e,f,g,h^	0.19 ^a,b,c^
		Ozonizer	1.34 ^l,ł,m,n,o,p,r^	0.34 ^a,b,c,d,e,f^	1.10 ^ij,k,l,ł,m,n,o^	0.32 ^a,b,c,d,e,f^
		RCI	1.65 ^o,p,r,s,t,u^	0.36 ^a,b,c,d,e,f,g^	2.12 ^u,w^	0.38 ^a,b,c,d,e,f,g^
	120 min	Fan	0.98 ^h,i,j,k,l,ł,m^	0.38 ^a,b,c,d,e,f,g^	0.95 ^h,i,j,k,l,ł^	0.39 ^a,b,c,d,e,f,g^
		Ozonizer	1.91 ^s,t,u,w^	0.52 ^a,b,c,d,e,f,g,h^	1.36 ^ł,m,n,o,p,r^	0.49 ^a,b,c,d,e,f,g,h^
		RCI	1.87 ^s,t,u,w^	0.51 ^a,b,c,d,e,f,g,h^	3.54 ^z^	0.55 ^a,b,c,d,e,f,g,h^

*—decrease in bacteria number below the detection limit, ^†^—time of action; PM (+)—with poultry manure; PM (−)—without poultry manure CFU–colony forming units; ^a,b,c,…^—values marked with different letters differ statistically significant, applies to the entire table.

**Table 3:** The final number of *Salmonella* spp. on eggshells contaminated with bacterial suspension of 10^5^ CFU/mL with or without the addition of poultry manure.

Variant		Temperature 4 °C		Temperature 20 °C	
		PM (−)	PM (+)	PM (−)	PM (+)
		Average [log CFU/egg] (±STD) *	Average [log CFU/egg] (±STD) *	Average [log CFU/egg] (±STD) *	Average [log CFU/egg] (±STD) *
Control		2.65 ^a,b^ (±0.21)	2.75 ^a^ (±0.20)	2.65 ^a,b^ (±0.21)	2.75 ^a^ (±0.20)
30 min ^†^	Fan	2.27 ^a,b,c,d^ (±0.17)	2.61 ^a,b^ (±0.18)	2.28 ^a,b,c,d^ (±0.17)	2.62 ^a,b^ (±0.18)
	Ozonizer	1.53 ^a,b,c,d,e,f^ (±0.10)	2.46 ^a,b,c^ (±0.17)	1.69 ^a,b,c,d,e,f^ (±0.11)	2.48 ^a,b,c^ (±0.17)
	RCI	1.37 ^b,c,d,e,f^ (±0.08)	2.45 ^a,b,c^ (±0.17)	1.29 ^c,d,e,f,g^ (±0.07)	2.43 ^a,b,c^ (±0.16)
60 min	Fan	1.89 ^a,b,c,d,e^ (±0.13)	2.54 ^a,b,c^ (±0.18)	1.91 ^a,b,c,d,e^ (±0.13)	2.53 ^a,b,c^ (±0.18)
	Ozonizer	0.66 ^e,f,g,h^ (±0.01)	2.36 ^a,b,c^ (±0.16)	0.82 ^e,f,g,h^ (±0.07)	1.57 ^a,b,c,d,e,,f^ (±0.16)
	RCI	0.71 ^e,f,g,h^ (±0.01)	2.35 ^a,b,c^ (±0.15)	0.07 ^g,h^ (±0.09)	1.53 ^a,b,c,d,e,f^ (±0.15)
120 min	Fan	0.96 ^d,e,f,g,h^ (±0.04)	2.37 ^a,b,c^ (±0.16)	1.30 ^c,d,e,f,g^ (±0.07)	2.38 ^a,b,c^ (±0.16)
	Ozonizer	0.52 ^f,g,h^ (±0.01)	2.16 ^a,b,c,d^ (±0.14)	0.01 ^g,h^ (±0.03)	0.01 ^g,h^ (±0.05)
	RCI	0.50 ^f,g,h^ (±0.01)	2.15 ^a,b,c,d^ (±0.17)	0.00 ^h^ (±0.00)	0.00 ^h^ (±0.00)

*—standard deviation, ^†^—time of action; PM (+)—with poultry manure; PM (−)—without poultry manure CFU–colony forming units; ^a,b,c,…^—values marked with different letters differ statistically significant, applies to the entire table.

**Table 4:** The final number of *Salmonella* spp. on eggshells contaminated with bacterial suspension of 10^8^ CFU/mL with or without the addition of poultry manure.

Variant		Temperature 4 °C		Temperature 20 °C	
		PM (−)	PM (+)	PM (−)	PM (+)
		Average [log CFU/egg] (±STD) *	Average [log CFU/egg] (±STD)	Average [log CFU/egg] (±STD)	Average [log CFU/egg] (±STD)
	Control	5.81 ^a,b^ (±0.51)	5.89 ^a^ (±0.50)	5.81 ^a,b^ (±0.51)	5.89 ^a^ (±0.50)
30 min ^†^	Fan	5.48 ^a,b,c,d^ (±0.47)	5.80 ^a,b^ (±0.49)	5.49 ^a,b,c,d^ (±0.47)	5.79 ^a,b^ (±0.49)
	Ozonizer	4.86 ^f,g,h^ (±0.41)	5.67 ^a,b,c,d^ (±0.48)	4.96 ^e,f,g^ (±0.42)	5.69 ^a,b,c^ (±0.48)
	RCI	4.83 ^f,g,h^ (±0.41)	5.63 ^a,b,c,d^ (±0.48)	4.75 ^f,g,h^ (±0.40)	5.63 ^a,b,c,d^ (±0.48)
60 min	Fan	5.21 ^d,e,f^ (±0.45)	5.70 ^a,b^ (±0.48)	5.22 ^c,d,e,f^ (±0.45)	5.70 ^a,b^ (±0.48)
	Ozonizer	4.47 ^h,i^ (±0.37)	5.55 ^a,b,c,d^ (±0.47)	4.71 ^g,h^ (±0.40)	5.57 ^a,b,c,d^ (±0.47)
	RCI	4.16 ^i,j^ (±0.34)	5.53 ^a,b,c,d^ (±0.47)	3.69 ^j^ (±0.29)	5.51 ^a,b,c,d^ (±0.46)
120 min	Fan	4.83 ^f,g,h^ (±0.41)	5.51 ^a,b,c,d^ (±0.46)	4.85 ^f,g,h^ (±0.41)	5.50 ^a,b,c,d^ (±0.46)
	Ozonizer	3.90 ^j^ (±0.32)	5.37 ^b,c,d,e^ (±0.45)	4.45 ^h,i^ (±0.37)	5.40 ^b,c,d,e^ (±0.45)
	RCI	3.93 ^j^ (±0.32)	5.38 ^b,c,d,e^ (±0.45)	2.27 ^k^ (±0.15)	5.34 ^b,c,d,e^ (±0.45)

*—standard deviation, ^†^—time of action; PM (+)—with poultry manure; PM (−)—without poultry manure CFU–colony forming units; ^a,b,c,…^—values marked with different letters differ statistically significant, applies to the entire table.

## Data Availability

The data presented in this study are available on request from the corresponding author.

## Supplementary Materials

The following supporting information can be downloaded at: https://www.mdpi.com/article/10.3390/foods11162452/s1, Table S1: The changes in the number of S. Enteritidis, S. Typhimurim, S. Virchow on eggshells contaminated with bacterial suspension of 103 CFU/mL with or without the addition of poultry manure; Table S2: The changes in the number of S. Enteritidis, S. Typhimurim, S. Virchow on eggshells contaminated with bacterial suspension of 105 CFU/mL with or without the addition of poultry manure; Table S3: The changes in the number of *S. enteritidis*, *S. typhimurim*, *S. virchow* on eggshells contaminated with bacterial suspension of 10^8^ CFU/mL with or without the addition of poultry manure.
