# Supplementary material for: Effect of Radiant Catalytic Ionization and Ozonation on Salmonella spp. on Eggshells
Source: Foods. 2022 Aug 14;11(16):2452. doi: 10.3390/foods11162452 (PMC9407475; doi:10.3390/foods11162452)
Supplement: Supplementary file 1 [file foods-11-02452-s001.zip › Grudlewska-Buda K. et al. - Table S1.pdf]

Table S1. The changes in the number of *S.* Enteritidis, *S.* Typhimurim, *S.* Virchow on eggshells contaminated with bacterial suspension of 10^3 CFU with or without the addition of poultry manure.

Variant			*S.* Enteritidis	*S.* Typhimurium	*S.* Virchow
			Average [log CFU/egg] *STD**	Average [log CFU/egg] *STD*	Average [log CFU/egg] *STD*
**4°C**	**Bacterial suspension 10^3 CFU**				
	Control		1.51 (±*0.09)*	1.59 (±*0.06)*	1.67 (±*0.10)*
	30 min. †	RCI	0.02 *(±0.06)*	0.21 (±*0.08)*	0.03 (±*0.07)*
		Ozonizer	0.26 (±*0.03)*	0.54 (±*0.04)*	0.23 (±*0.04)*
		Fan	0.58 (±*0.00)*	0.85 (±*0.01)*	0.30 (±*0.04)*
	60 min.	RCI	0.00 (±*0.00)*	0.00 (±*0.00)*	0.00 (±*0.00)*
		Ozonizer	0.00 (±*0.00)*	0.00 (±*0.00)*	0.00 (±*0.00)*
		Fan	0.00 (±*0.00)*	0.00 (±*0.00)*	0.00 (±*0.00)*
	120 min.	RCI	0.00 (±*0.00)*	0.00 (±*0.00)*	0.00 (±*0.00)*
		Ozonizer	0.00 (±*0.00)*	0.00 (±*0.00)*	0.00 (±*0.00)*
		Fan	0.00 (±*0.00)*	0.00 (±*0.00)*	0.00 (±*0.00)*
	**Bacterial suspension 10^3 and poultry manure**				
	Control		1.60 (±*0.10)*	1.67 (±*0.07)*	1.74 (±*0.09)*
	30 min.	RCI	0.23 (±*0.04)*	0.38 (±*0.06)*	0.22 (±*0.06)*
		Ozonizer	0.54 (±*0.01)*	0.74 (±*0.02)*	0.56 (±*0.03)*
		Fan	0.92 (±*0.03)*	1.10 (±*0.01)*	0.86 (±*0.00)*
	60 min.	RCI	0.00 (±*0.00)*	0.00 (±*0.00)*	0.00 (±*0.00)*
		Ozonizer	0.00 (±*0.00)*	0.00 (±*0.00)*	0.00 (±*0.00)*
		Fan	0.00 (±*0.00)*	0.00 (±*0.00)*	0.00 (±*0.00)*
	120 min.	RCI	0.00 (±*0.00)*	0.00 (±*0.00)*	0.00 (±*0.00)*
		Ozonizer	0.00 (±*0.00)*	0.00 (±*0.00)*	0.00 (±*0.00)*
		Fan	0.00 (±*0.00)*	0.00 (±*0.00)*	0.00 (±*0.00)*
**20°C**	**Bacterial suspension 10^3 CFU**				
	Control		1.51 (±*0.09)*	1.59 (±*0.06)*	1.67 (±*0.10)*
	30 min.	RCI	0.00 (±*0.00)*	0.09 (±*0.09)*	0.00 (±*0.00)*
		Ozonizer	0.33 (±*0.02)*	0.59 (±*0.04)*	0.32 (±*0.00)*
		Fan	0.57 (±*0.00)*	0.88 (±*0.00)*	0.27 (±*0.00)*
	60 min.	RCI	0.00 (±*0.00)*	0.00 (±*0.00)*	0.00 (±*0.00)*
		Ozonizer	0.00 (±*0.00)*	0.00 (±*0.00)*	0.00 (±*0.00)*
		Fan	0.00 (±*0.00)*	0.00 (±*0.00)*	0.00 (±*0.00)*
	120 min.	RCI	0.00 (±*0.00)*	0.00 (±*0.00)*	0.00 (±*0.00)*
		Ozonizer	0.00 (±*0.00)*	0.00 (±*0.00)*	0.00 (±*0.00)*
		Fan	0.00 (±*0.00)*	0.00 (±*0.00)*	0.00 (±*0.00)*
	**Bacterial suspension 10^3 and poultry manure**				
	Control		1.60 (±*0.01)*	1.67 (±*0.07)*	1.74 (±*0.09)*
	30 min.	RCI	0.15 (±*0.05)*	0.33 (±*0.06)*	0.11 (±*0.07)*

Variant		*S.* Enteritidis	*S.* Typhimurium	*S.* Virchow
		**Average [log CFU/egg]** *STD**	**Average [log CFU/egg]** *STD*	**Average [log CFU/egg]** *STD*
	Ozonizer	0.61 (*±0.00)*	0.80 (*±0.02)*	0.66 (*±0.02)*
	Fan	0.94 (*±0.03)*	1.10 (*±0.01)*	0.90 (*±0.00)*
60 min.	RCI	0.00 (*±0.00)*	0.00 (*±0.00)*	0.00 (*±0.00)*
	Ozonizer	0.00 (*±0.00)*	0.00 (*±0.00)*	0.00 (*±0.00)*
	Fan	0.00 (*±0.00)*	0.00 (*±0.00)*	0.00 (*±0.00)*
120 min.	RCI	0.00 (*±0.00)*	0.00 (*±0.00)*	0.00 (*±0.00)*
	Ozonizer	0.00 (*±0.00)*	0.00 (*±0.00)*	0.00 (*±0.00)*
	Fan	0.00 (*±0.00)*	0.00 (*±0.00)*	0.00 (*±0.00)*

* - standard deviation, † - time of action; CFU – colony forming units
