# Supplementary material for: Effect of Radiant Catalytic Ionization and Ozonation on Salmonella spp. on Eggshells
Source: Foods. 2022 Aug 14;11(16):2452. doi: 10.3390/foods11162452 (PMC9407475; doi:10.3390/foods11162452)
Supplement: Supplementary file 1 [file foods-11-02452-s001.zip › Grudlewska-Buda K. et al. - Table S2.pdf]

**Table S2.** The changes in the number of *S.* Enteritidis, *S.* Typhimurim, *S.* Virchow on eggshells contaminated with bacterial suspension of $10^5$ CFU with or without the addition of poultry manure.

| Variant | | | *S.* Enteritidis | *S.* Typhimurium | *S.* Virchow |
|---|---|---|---|---|---|
| | | | **Average [log CFU/egg]** *(STD)** | **Average [log CFU/egg]** *(STD)* | **Average [log CFU/egg]** *(STD)* |
| **4°C** | **Bacterial suspension $10^5$ CFU** | | | | |
| | Control | | 2.74 (±*0.20)* | 2.34 (±*0.22)* | 2.88 (±*0.21)* |
| | 30 min. + | RCI | 1.52 (±*0.07)* | 1.30 (±*0.11)* | 1.30 (±*0.05)* |
| | | Ozonizer | 1.63 (±*0.08)* | 1.41 (±*0.13)* | 1.55 (±*0.08)* |
| | | Fan | 2.42 (±*0.20)* | 2.04 (±*0.19)* | 2.36 (±*0.16)* |
| | 60 min. | RCI | 0.78 (±*0.00)* | 0.45 (±*0.04)* | 0.89 (±*0.00)* |
| | | Ozonizer | 0.74 (±*0.00)* | 0.55 (±*0.04)* | 0.69 (±*0.01)* |
| | | Fan | 2.25 (±*0.14)* | 1.92 (±*0.18)* | 1.51 (±*0.07)* |
| | 120 min. | RCI | 0.59 (±*0.02)* | 0.04 (±*0.00)* | 0.88 (±*0.00)* |
| | | Ozonizer | 0.91 (±*0.01)* | 0.06 (±*0.00)* | 0.58 (±*0.02)* |
| | | Fan | 1.55 (±*0.07)* | 1.40 (±*0.13)* | 0.07 (±*0.09)* |
| | **Bacterial suspension $10^5$ and poultry manure** | | | | |
| | Control | | 2.81 (±*0.20)* | 2.51 (±*0.18)* | 2.92 (±*0.21)* |
| | 30 min. | RCI | 2.51 (±*0.17)* | 2.28 (±*0.15)* | 2.55 (±*0.17)* |
| | | Ozonizer | 2.52 (±*0.17)* | 2.30 (±*0.16)* | 2.57 (±*0.17)* |
| | | Fan | 2.68 (±*0.19)* | 2.43 (±*0.17)* | 2.74 (±*0.19)* |
| | 60 min. | RCI | 2.39 (±*0.16)* | 2.20 (±*0.13)* | 2.45 (±*0.16)* |
| | | Ozonizer | 2.41 (±*0.16)* | 2.22 (±*0.15)* | 2.44 (±*0.16)* |
| | | Fan | 2.58 (±*0.18)* | 2.36 (±*0.17)* | 2.66 (±*0.18)* |
| | 120 min. | RCI | 2.19 (±*0.20)* | 2.02 (±*0.18)* | 2.25 (±*0.14)* |
| | | Ozonizer | 2.14 (±*0.13)* | 2.05 (±*0.13)* | 2.28 (±*0.15)* |
| | | Fan | 2.44 (±*0.16)* | 2.22 (±*0.15)* | 2.47 (±*0.16)* |
| **20°C** | **Bacterial suspension $10^5$ CFU** | | | | |
| | Control | | 2.74 (±*0.19)* | 2.34 (±*0.22)* | 2.88 (±*0.21)* |
| | 30 min. | RCI | 1.44 (±*0.06)* | 1.24 (±*0.11)* | 1.18 (±*0.04)* |
| | | Ozonizer | 1.78 (±*0.10)* | 1.49 (±*0.14)* | 1.79 (±*0.10)* |
| | | Fan | 2.43 (±*0.16)* | 2.05 (±*0.19)* | 2.36 (±*0.16)* |
| | 60 min. | RCI | 0.04 (±*0.08)* | 0.12 (±*0.00)* | 0.04 (±*0.19)* |
| | | Ozonizer | 1.38 (±*0.06)* | 1.04 (±*0.09)* | 0.04 (±*0.07)* |
| | | Fan | 2.23 (±*0.14)* | 1.92 (±*0.18)* | 1.58 (±*0.08)* |
| | 120 min. | RCI | 0.00 (±*0.00)* | 0.00 (±*0.00)* | 0.00 (±*0.00)* |
| | | Ozonizer | 0.00 (±*0.00)* | 0.04 (±*0.08)* | 0.00 (±*0.00)* |
| | | Fan | 1.59 (±*0.08)* | 1.38 (±*0.13)* | 0.93 (±*0.01)* |
| | **Bacterial suspension $10^5$ and poultry manure** | | | | |
| | Control | | 2.81 (±*0.20)* | 2.51 (±*0.18)* | 2.92 (±*0.21)* |
| | 30 min. | RCI | 2.49 (±*0.17)* | 2.27 (±*0.16)* | 2.53 (±*0.17)* |
| | | Ozonizer | 2.54 (±*0.17)* | 2.32 (±*0.16)* | 2.60 (±*0.18)* |
| | | Fan | 2.68 (±*0.19)* | 2.42 (±*0.17)* | 2.74 (±*0.19)* |
| | 60 min. | RCI | 2.37 (±*0.16)* | 2.18 (±*0.15)* | 0.04 (±*0.16)* |

| Variant | | *S.* Enteritidis | *S.* Typhimurium | *S.* Virchow |
|---|---|---|---|---|
| | | **Average [log CFU/egg] *(STD)**** | **Average [log CFU/egg] *(STD)*** | **Average [log CFU/egg] *(STD)*** |
| | Ozonizer | 2.44 (±*0.16)* | 2.24 (±*0.15)* | 0.04 (±*0.16)* |
| | Fan | 2.58 (±*0.18)* | 2.36 (±*0.17)* | 2.66 (±*0.18)* |
| 120 min. | RCI | 0.00 (±*0.00)* | 0.00 (±*0.00)* | 0.00 (±*0.00)* |
| | Ozonizer | 0.00 (±*0.00)* | 0.04 (±*0.14)* | 0.00 (±*0.00)* |
| | Fan | 2.45 (±*0.16)* | 2.22 (±*0.15)* | 2.48 (±*0.17)* |

* - standard deviation, † - time of action; CFU – colony forming units
