# Supplementary material for: Effect of Radiant Catalytic Ionization and Ozonation on Salmonella spp. on Eggshells
Source: Foods. 2022 Aug 14;11(16):2452. doi: 10.3390/foods11162452 (PMC9407475; doi:10.3390/foods11162452)
Supplement: Supplementary file 1 [file foods-11-02452-s001.zip › Grudlewska-Buda K. et al. - Table S3.pdf]

Table S3. The changes in the number of *S.* Enteritidis, *S.* Typhimurim, *S.* Virchow on eggshells contaminated with bacterial suspension of 10^8 CFU with or without the addition of poultry manure.

Variant			*S.* Enteritidis	*S.* Typhimurium	*S.* Virchow
			**Average [log CFU/egg] *(STD)****	**Average [log CFU/egg] *(STD)***	**Average [log CFU/egg] *(STD)***
**4°C**	**Bacterial suspension 10^8 CFU**				
	Control		5.80 (*±0.51)*	5.67 (*±0.51)*	5.95 *(±0.50)*
	30 min. +	RCI	4.82 (*±0.41)*	4.77 (*±0.42)*	4.89 (*±0.40)*
		Ozonizer	4.85 (*±0.41)*	4.82 (*±0.43)*	4.92 (*±0.40)*
		Fan	5.55 (*±0.48)*	5.43 (*±0.49)*	5.47 (*±0.46)*
	60 min.	RCI	4.16 (*±0.34)*	4.10 (*±0.35)*	4.21 (*±0.33)*
		Ozonizer	4.58 (*±0.38)*	4.58 (*±0.40)*	4.26 (*±0.33)*
		Fan	5.43 (*±0.47)*	5.34 (*±0.48)*	4.85 (*±0.39)*
	120 min.	RCI	3.91 (*±0.32)*	3.82 (*±0.33)*	4.06 (*±0.31)*
		Ozonizer	3.90 (*±0.31)*	3.88 (*±0.33)*	3.91 (*±0.40)*
		Fan	5.05 (*±0.43)*	5.07 (*±0.45)*	4.36 (*±0.34)*
	**Bacterial suspension 10^8 and poultry manure**				
	Control		5.88 (*±0.50)*	5.81 (*±0.52)*	5.98 (*±0.48)*
	30 min. +	RCI	5.63 (*±0.48)*	5.65 (*±0.51)*	5.62 (*±0.46)*
		Ozonizer	5.65 (*±0.48)*	5.68 (*±0.51)*	5.68 (*±0.45)*
		Fan	5.80 (*±0.49)*	5.76 (*±0.52)*	5.83 (*±0.47)*
	60 min.	RCI	5.49 (*±0.46)*	5.55 (*±0.50)*	5.55 (*±0.47)*
		Ozonizer	5.53 (*±0.47)*	5.57 (*±0.50)*	5.55 (*±0.47)*
		Fan	5.69 (*±0.48)*	5.69 (*±0.51)*	5.74 (*±0.46)*
	120 min.	RCI	5.33 (*±0.45)*	5.40 (*±0.48)*	5.39 (*±0.42)*
		Ozonizer	5.35 (*±0.45)*	5.37 (*±0.48)*	5.38 (*±0.42)*
		Fan	5.49 (*±0.46)*	5.54 (*±0.49)*	5.48 (*±0.43)*
**20°C**	**Bacterial suspension 10^8 CFU**				
	Control		5.80 (*±0.51)*	5.67 (*±0.51)*	5.95 *(±0.50)*
	30 min. +	RCI	4.75 (*±0.40)*	4.71 (*±0.42)*	4.80 *(±0.39)*
		Ozonizer	4.95 (*±0.42)*	4.90 (*±0.43)*	5.03 *(±0.41)*
		Fan	5.55 (*±0.48)*	5.44 (*±0.49)*	5.48 *(±0.46)*
	60 min.	RCI	3.70 (*±0.30)*	3.71 (*±0.32)*	3.65 *(±0.27)*
		Ozonizer	4.75 (*±0.40)*	4.71 (*±0.42)*	4.65 *(±0.37)*
		Fan	5.42 (*±0.47)*	5.33 (*±0.48)*	4.91 *(±0.40)*
	120 min.	RCI	2.10 (*±0.14)*	2.75 (*±0.22)*	1.95 *(±0.10)*
		Ozonizer	4.44 (*±0.37)*	4.34 (*±0.38)*	4.56 *(±0.36)*
		Fan	5.08 (*±0.43)*	5.05 (*±0.45)*	4.43 *(±0.35)*
	**Bacterial suspension 10^8 and poultry manure**				
	Control		5.88 (*±0.50)*	5.81 (*±0.52)*	5.98 *(±0.48)*
	30 min. +	RCI	5.62 (*±0.48)*	5.65 (*±0.51)*	5.62 *(±0.45)*
		Ozonizer	5.67 (*±0.48)*	5.69 (*±0.51)*	5.70 *(±0.45)*
		Fan	5.79 (*±0.49)*	5.76 (*±0.52)*	5.83 *(±0.47)*

Time	Variant	*S.* Enteritidis	*S.* Typhimurium	*S.* Virchow
		**Average [log CFU/egg] *(STD)****	**Average [log CFU/egg] *(STD)***	**Average [log CFU/egg] *(STD)***
60 min.	RCI	5.50 (*±0.46)*	5.53 (*±0.49)*	5.52 *(±0.44)*
	Ozonizer	5.56 (*±0.47)*	5.59 (*±0.50)*	5.58 *(±0.44)*
	Fan	5.69 (*±0.48)*	5.69 (*±0.51)*	5.73 *(±0.46)*
120 min.	RCI	5.30 (*±0.44)*	5.38 (*±0.48)*	5.36 *(±0.42)*
	Ozonizer	5.39 (*±0.45)*	5.40 (*±0.48)*	5.42 *(±0.43)*
	Fan	5.50 (*±0.46)*	5.53 (*±0.49)*	5.48 *(±0.43)*

* - standard deviation, † - time of action; CFU – colony forming units
